# The effect of extended lactation on parameters of Wood’s model of lactation curve in dairy Simmental cows

**DOI:** 10.5713/ajas.20.0347

**Published:** 2020-10-13

**Authors:** Tomáš Kopec, Gustav Chládek, Daniel Falta, Josef Kučera, Milan Večeřa, Oto Hanuš

**Affiliations:** 1 Department of Animal Breeding, Mendel University in Brno (FA), Zemědělská 1, 613 00 Brno, Czech Republic; 2 Czech Moravian Breeders Association, Benešovská 123, 252 09 Hradištko, Czech Republic; 3 Dairy Research Institute Ltd., 160 00 Prague, Czech Republic

**Keywords:** Dairy Cows, Simmental Breed, Wood’s Function, Lactation, Milk Yield

## Abstract

**Objective:**

This study was focused on the estimation of parameters of Wood’s model and description of the lactation curve using the cows which were lactated over 24 months on the first lactation.

**Methods:**

The database included 1,333 pure-bred dairy Simmental primiparous cows which lactated for 24 months (732 days). The initial dataset entering the procedure of assessment of parameters of Wood’s function included 35,826 milk yield records. Milk yield was recorded throughout lactation, with the earliest record taken on day 6 and the latest on day 1,348 of lactation. This dataset was used for the assessment of parameters *a*, *b*, *c* of Wood’s model using the non-linear statistical procedure. These parameters were estimated for different length of lactation. The assessed parameters were used for calculation of some characteristics of lactation curves.

**Results:**

The lowest value of *a* parameter (15.2317) of Wood’s model of lactation curve was found out in lactations up to 305 days long, contrary to *b* and *c* parameters which were highest in those lactations (0.1029 and 0.0015, respectively). The maximum value of *a* parameter (17.4329) was found out in lactations up to 640 days long, unlike *b* and *c* parameters which were minimal in those lactations (0.0603 and 0.0010, respectively).

**Conclusion:**

It can be concluded that the parameters of Wood’s model and the shape of lactation curve are changing with the growing number of milk yield records. Also, the assessed parameters revealed a significant milk production potential after 305 days of lactation.

## INTRODUCTION

Czech dairy Simmental cattle belongs to the family of dual-purpose breeds of the phylogenetic group of “Simmental type breed” as well as other breeds derived from Simmental cattle such as Fleckvieh, Montbéliarde and Pezzata Rossa. The description of lactation curve is still current in livestock research and was analysed by others [1–3]. Lactations are described for many reasons, for example estimating of genetic parameters, analysing of genomic evaluation and comparison of data from milking system and test-day records [4–6]. The conventional length of a lactation period is 305 days. Lactation is terminated either spontaneously or deliberately by the breeder 60 days prior to parturition (provided the cow had conceived at the optimal period). Nowadays, on the one hand milk production of cows is increasing but on the other hand their reproductive performance is deteriorating. Consequently, cows conceive later and at the end of 305-day lactation period do not reach the point of gestation when they should be dried off (which is about 60 days prior to parturition). Their milk production is still relatively high and profitable. With the present purchase milk price of 35 cents per litre and production costs of 7.70 € per day in the Czech Republic, the limit of profitability is about 22 kg of milk per day. This limit is easily exceeded by most cows. Consequently, milking periods longer than 305 days are becoming a common phenomenon. Therefore, it is essential to study and define parameters of extended lactation curves. Extended lactation periods were analysed by others [7–11].

The course of lactation is well described by Wood’s mathe matical function [12]. This model is favoured for its simplicity and accuracy of description [13,14]. Wood’s model is also used to describe lactation in other farm animals, e.g. Bilgin et al [15] used it to assess the course of lactation in sheep. It is an incomplete gamma function, where milk yield per day is the dependant variable and day of lactation is the explanatory variable. The model includes three parameters (*a*, *b*, *c*), which are assessed; *a* parameter represents the initial milk yield, *b* and *c* parameters relate to the rise or decline of a lactation curve [16]. Phenotypic study of lactation curve was described by Farhangfar and Naeemipour [17]. The Wood’s incomplete gamma function was one of the earliest popular models conceived for the lactation [9]. Wood’s model is non-linear three-parameter model and such models could be more suitable for describing extended lactations, especially for use in practice due to the ratio of simplicity and accuracy of description. This model is empirical, because it establishes only relationship between variables [9,11]. In case of using more complex analysis (estimation of breeding values, linear models with more effects) there are other models with more parameters (Legendre polynomials, etc.). Dematawewa et al [9,11,14] compared different mathematical models including Wood’s model.

As mentioned above, lactations are getting longer, and it is necessary to determine the effect of extended lactations on parameters of Wood’s model if we want to describe lactation curves accurately.

## MATERIALS AND METHODS

The dataset (Table 1) used for assessment of parameters of Wood’s function included information on milk yield of primiparous Czech dairy cows of Simmental breed (SB) cows (minimum of 75% of SB in genotype). The database was provided by Czech-Moravian breeders corporation and contained milk-recording data on cows born between 1995 and 2012 with the minimal length of lactation 24 months (732 days). The overall number of animals was 1,333. Milk yield of each cow was recoded 24 (minimum) to 30 times (maximum) with the average of 27 recordings per animal (the number was limited by the official methodology of milk-recoding in the Czech Republic by ICAR—International Committee for Animal Recording). The initial dataset entering the procedure of assessment of parameters of Wood’s function included 35,826 milk yield records. Milk yield was recorded throughout lactation, with the earliest record taken on day 6 and the latest on day 1,348 of lactation. The average milk yield was 16.34 kg (sd 5.39), ranging from 3 kg to 64 kg of milk per test recording.

This dataset was used for the assessment of parameters *a*, *b*, *c* of Wood’s model using the non-linear procedure NLIN in SAS 9.1. The applied Wood’s model can be expressed as:

(1) $$\text{y}={\text{at}}^{\text{b}}{\text{e}}^{-\text{ct}},$$

where *a*, *b*, *c* are the assessed parameters of the function; *y* is milk yield per day (kg); *t* day of lactation when the milk yield was recorded; *e* is the base of the natural logarithm.

This function was applied to assess *a*, *b*, *c* parameters. First, the analysed dataset was unrestricted —it included data on lactations with the greatest possible number of milk records (maximum 30 records). Then the dataset was restricted—it only included data on lactations up to 732 days long (maximum 24 records) and again, the parameters of Wood’s function were estimated. In this manner, the analysis was repeated, until the dataset was restricted down to lactations up to 305 days long (maximum 10 records).

As a result, we got 15 different coefficients *a*, *b*, *c* which were calculated using the same database, but each time restricted in the manner described above (the number of records ranging from 10 to 24). The assessed parameters were used for calculation of some characteristics of lactation curves (see Table 2) and, lactation curves were constructed. Milk yields per a 305-day lactation and per 100-day sections of lactation (day 1 to 100, … up to day 601 to 700) and subsequently their respective indices of persistency (IP2:1, IP3:1, IP4:1, IP5:1, IP6:1, IP7:1) were calculated.

Table 1 presents some significant characteristics of lacta tion curve: *t* is the day of maximum milk yield in the lactation, *y_m_* is the maximum yield on day *t*, *r* is a coefficient describing the rate of decline of the lactation curve between its top and end, *a*, *b*, *c* are assessed parameters of Wood’s model. These parameters were used to form the shape of the lactation curve depending to the length of lactation. At the same time, total and partial 100-day milk yields were calculated and indices of persistency expressed as: IP = (milk yield per 101 to 200 days, 201 to 300 days…601 to 700 days of lactation/milk yield per 0 to 100 days of lactation)×100.

## RESULTS AND DISCUSSION

There were 15 variations of Wood’s model and its parameters differing in the number of milk yield records entering the analysis (Table 3). All variants of Wood’s model were statistically significant and suitable for describing the lactation curve (F-test of model was in all cases F<0.0001).

**Figure 1:**
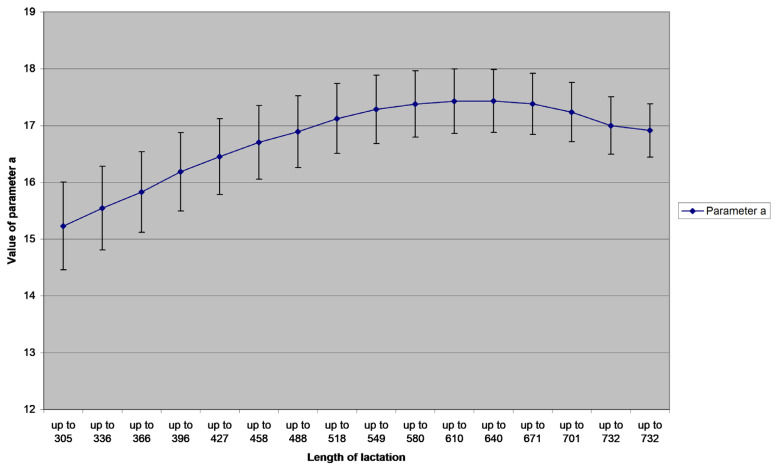
Mean values and their standard errors (+/− 2.96 standard error) of the assessed parameter *a* of Wood’s model for different lengths of lactation (in days).

**Figure 2:**
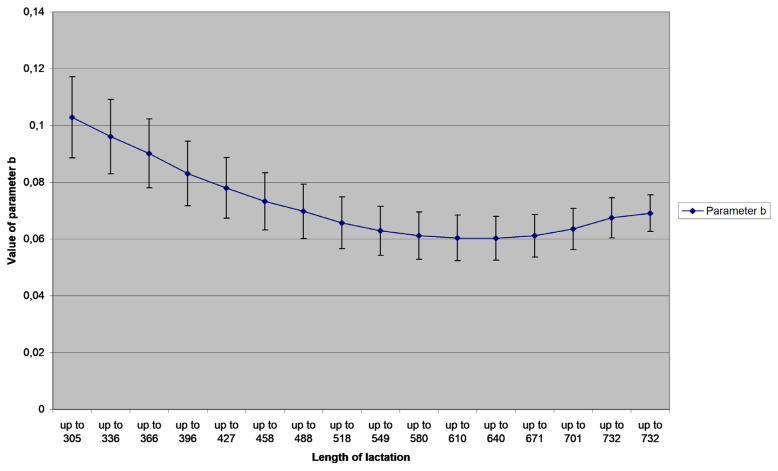
Mean values and their standard errors (+/− 2.96 standard error) of the assessed parameter *b* of Wood’s model for different lengths of lactation (in days).

**Figure 3:**
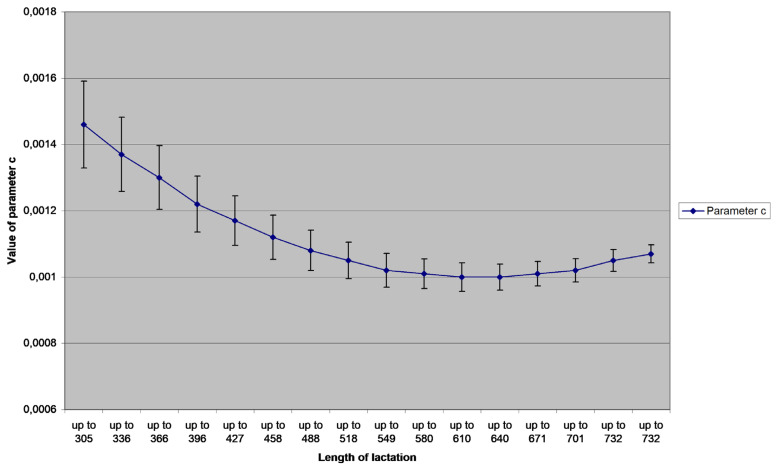
Mean values and their standard errors (+/− 2.96 standard error) of the assessed parameter *c* of Wood’s model for different lengths of lactation (in days).

The variability of parameters (with the 95% confidence interval) related to the amount of information (number of records) is portrayed in Figure 1 to 3. Value of *a* parameter grows with the growing number of milk yield records up to the length of lactation 640 days (21 records), and then it slightly decreases. Both *b* and *c* parameters decrease with the growing number of records; *b* decreases again until test day 21 (21 records), *c* stopped decreasing between the test days 20 and 21. The graph reveals that parameter *a* is significantly different in the group 305 to 366 days and group 549 to 701 days of lactation; the *a* value is significantly lower in the first group. Parameter *b* is significantly lower from 518 days on, compared to the group up to 366 days of lactation. Parameter *c* is significantly lower from 549 days on, compared to the group up to 396 days of lactation.

**Figure 4:**
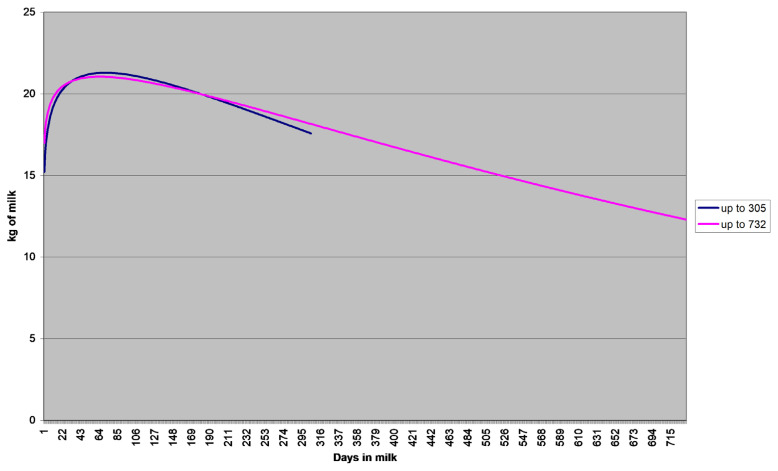
Shape of lactation curve according to the length of lactation (amount of information). Shorter curve indicates average daily milk yield up to 305 days of lactation and longer curve up to 732 days of lactation.

Values of these parameters reveal differences in shapes of lactation curves caused by different numbers of records. Figure 4 presents two lactation curves shaped by the assessed parameters of Wood’s model. The first relates to the cows with 10 to 11 milk yield records and the second to the cows with the maximum number of records registered in the official national milk-recording in the Czech Republic, i.e. 30 milk yield records. Both curves show a very mild decrease in milk yield during the standard 305-day lactation. The curve representing the extended lactation shows even slighter decrease and the milk yield on day 305 does not fall below 18 kg.

Parameters of Wood’s function in cattle milk production were assessed by Abdelsayed et al [18]. They found out the following values: *a* = 18.32, *b* = 0.1428, and *c* = 0.0040. Similar values were determined in Holstein cattle (*a* = 12.1, *b* = 0.285, and *c* = 0.00328) [19]. The parameters of Wood’s model found in Jersey cattle [14] were following: *a* = 15.46, *b* = 0.12, and *c* = 0.09. Třináctý et al [13] and Kopec et al [20] applied Wood’s function in assessment of lactation curves in Czech Simmental dairy cattle with similar results. The parameters presented by Třináctý et al [13] were a bit lower which was due to the lower overall milk yield in the tested group of cows. Parameters in both publications corresponded with our values. In other study was assessed parameters of Wood’s model in dairy cattle in Italy [21] with the following results: *a* = 11.17, *b* = 0.267, and *c* = 0.00564. Mentioned parameters was assessed also by Polish Holstein [5], respectively Jersey in Turkey [14].

Dematawewa et al [9] studied the differences in parame ters of Wood’s model related to different lengths of lactation. They compared data on Holstein cows in the USA with the length of lactation either up to 305 days or 1,000 days. Parameters of Wood’s model for standard, 305-day lactations were: *a* = 15.6862, *b* = 0.2081, *c* = 0.002 and for extended lactation: *a* = 17.8808, *b* = 0.1616, *c* = 0.00107. Their results confirmed our findings that *a* parameter grows with the growing length of lactation while *b* and *c* parameters tend to decrease.

Table 4 shows some characteristics of lactation calculated using the assessed parameters. The cows lactating longer (with more milk yield records) reached the maximum production earlier than the cows lactating for a shorter period (maximum yield reached 70 days in lactation for the cows with 10 milk records and 60 days for the cows with 21 records). Cows with more than 21 records reached the maximum day slightly later. The maximum milk yield was almost identical in all groups and ranged from 21.02 kg in the group with 19 to 22 milk yield records to 21.29 in the group with 10 records. Zamani et al [22] reported average milk yield 25.09 kg. These values suggested that the maximum yield was slightly overvalued in long lactations with fewer milk yield records, and there was also about a 10-day shift in the day when the maximum milk yield was reached (maximum day).

The maximum day and the maximum milk yield and their relation to the length of lactation were determined by Pollott [23]. For a 305-day lactation the maximum milk yield of 28.5 kg was reached on day 34.2 of lactation. In lactations extended up to 370 or 440 days the maximum was reached 34.6 or 35.9 days, respectively, with the milk yield 29.2 or 29.3 kg milk, respectively. Steri et al [11] calculated the maximum milk yield of 31 kg recorded on day 63 in the normal, 305-day lactation and maximum of 30.7 kg of milk on day 71 of the extended, 1,000-day lactation. Dematawewa et al [9] determined the maximum yield of 33.35 kg of milk recorded on day 102 of the normal, 305-day lactation in Holstein cows and 34.22 kg of milk recorded on day 151 of the extended, 1,000-day lactation. Our results are most like those of Steri et al [11]. The differences reported in other studies may be explained by different breeds and farming conditions. Different values of peak yield and day of peak was described by Atashi et al [1,2,5,7,14].

The total milk yield per 305-day lactation was 6,058.48 kg and per 732-day was 12,530.69 kg of milk. The maximum length of lactation was 732 days, which is 2.4 times the length of the normal, 305-day lactation. The estimated total milk yield (12,530.69 kg) was 2.07 times greater than the estimated milk yield per 305 days. The average milk yield would be 19.86 kg of milk per day in a 305-day lactation and 17.12 kg of milk per day in a maximum lactation.

Vargas et al [24] described a milk yield of 5,310 kg per 305- day lactation and 8,409 kg per 547-day lactation. 5,313 kg of milk was produced in 305 days and the rest (3,096 kg, i.e. 37% of the total milk yield) was produced in the period between 306 and 547 days. Haile-Mariam and Goddard [10] reported the total milk yield 6,517 kg and 10,027 kg per 305-day and 545-day lactations, respectively. Wall et al [25] studied the effect of length of lactation on greenhouse gas emission. Ten percent of cows with the lowest milk yield produced 6,044 kg milk per 305-day lactation and 8,652 kg per 440-day lactation. Ten percent of best producing cows produced 13,673 kg and 14,867 kg of milk per 305 or 440 days, respectively. Abdelsayed et al [18] found out the milk yield of 8,887 kg produced in the period between 305 to 610 days, with the range 2,891 to 19,631 kg milk. Holstein cattle from various countries reached different milk yield per lactation [4,5,17].

Table 4 shows that the milk yield per partial 100-day peri ods decreased with progressing lactation and the indices of persistence, based on the partial milk yields, copied the tendency. The milk yield per period between 601 to 700 days made up about 65% of milk yield per period 0 to 100 days and about 10% of the total milk yield per 732 days. The index of persistency IP2:1 came close to 1 in extremely long lactations which means that the milk production in the period between 101 to 200 days continued to be almost as high as in the first 100 days of lactation. The value of index of persistency gradually decreases by 7% from IP2:1 = 98.62 to IP7:1 = 64.49.

Cankaya et al [16] determined IP2:1 = 96.1, Kopec et al [20] IP2:1 = 74.59 to 89.71 in Czech Simmental dairy cattle, Třináctý et al [13] assessed IP2:1 between 74.2 and 77.2. Our values of IP2:1 were considerably higher because the initial dataset included the data on extended lactations (up to 732 days) in which milk yield started to decline later in lactation. The values of IP3:1 to IP7:1 confirm this tendency.

## CONCLUSION

It can be concluded that the parameters of Wood’s model and the shape of lactation curve are changing with the growing number of milk yield records. Also, the assessed parameters revealed a significant milk production potential after 305 days of lactation; cows are able to produce almost the same amount of milk in the period between 305 and 732 days of lactation as they produce in the first 305 days. Above mentioned facts suggest that extended lactations are significant. It is necessary to collect data on milk production (milk-recording) beyond the normal length of lactation (305 days) and incorporate the new information into the genetic evaluation of milk production not only in Czech Simmental dairy cows. Famers can use a knowledge of extended lactation for estimating the milk yield on the end of lactation and use it in managing of conceiving of high milking cows. According our results, it is possible to conceive cows after ending (decreasing) the impact of negative energy balance of high milking cows.

## Figures and Tables

**Table 1:** Description of primary dataset

Items	Min	Max	Average
Brith year	1995	2012	-
Days in milk	6	1,348	811.2
No. of milk records per animal	24	30	27
Milk yield (kg)	3	64	16.34

**Table 2:** Calculation of some characteristics of lactation curve

Value	Formula
t	b/c
ym	a(b/c)^b^e^−b^
r	b/((t+305)/2) − c

t, day of lactation; y_m_, maximum daily milk yield during lactation; r, coefficient describing the rate of decline of the lactation curve between its top and end; a, b, c, parameters of Wood’s model.

**Table 3:** The effect of extended lactation on *a*, *b*, and *c* parameters of Wood’s model of lactation curve

Length of lactation (days)	Parameter a	Parameter b	Parameter c
up to 305	15.2317	0.1029	0.0015
up to 336	15.5457	0.0961	0.0014
up to 366	15.8302	0.0902	0.0013
up to 396	16.1852	0.0831	0.0012
up to 427	16.4543	0.0780	0.0012
up to 458	16.7037	0.0733	0.0011
up to 488	16.8936	0.0698	0.0011
up to 518	17.1234	0.0657	0.0011
up to 549	17.2846	0.0629	0.0010
up to 580	17.3794	0.0612	0.0010
up to 610	17.4283	0.0604	0.0010
up to 640	17.4329	0.0603	0.0010
up to 671	17.3811	0.0612	0.0010
up to 701	17.2370	0.0636	0.0010
up to 732	16.9995	0.0675	0.0011

**Table 4:** Characteristics of lactation according to the assessed parameters of Wood’s model

Length of lactation (amount of information)	t	y_m_	r	Total milk yield	Milk yield, day 1–100	Milk yield, day 101–200	Milk yield, day 201–300	Milk yield, day 301–400	Milk yield, day 401–500	Milk yield, day 501–600	Milk yield, day 601–700	IP21	IP31	IP41	IP51	IP61	IP71
up to 305	70.48	21.29	−0.000912	6,058.48	2,060.75	2,044.77	1,864.87	-	-	-	-	99.22	90.49	-	-	-	-
up to 336	70.15	21.25	−0.000858	6,609.81	2,060.74	2,044.68	1,874.94	-	-	-	-	99.22	90.98	-	-	-	-
up to 366	69.38	21.20	−0.000818	7,128.31	2,060.79	2,043.01	1,880.73	-	-	-	-	99.14	91.26	-	-	-	-
up to 396	68.11	21.15	−0.000775	7,640.10	2,061.15	2,040.43	1,886.40	-	-	-	-	98.99	91.52	-	-	-	-
up to 427	66.67	21.12	−0.000750	8,149.89	2,061.80	2,037.44	1,888.02	-	-	-	-	98.82	91.57	83.65	-	-	-
up to 458	65.45	21.09	−0.000724	8,656.55	2,062.52	2,035.57	1,891.08	1,733.38	-	-	-	98.69	91.69	84.04	-	-	-
up to 488	64.63	21.08	−0.000702	9,143.40	2,063.57	2,035.23	1,894.86	1,741.73	-	-	-	98.63	91.82	84.40	-	-	-
up to 518	62.57	21.04	−0.000693	9,593.66	2,063.58	2,030.24	1,891.83	1,741.73	1,594.39	-	-	98.38	91.68	84.40	77.26	-	-
up to 549	61.67	21.03	−0.000677	10,076.07	2,064.90	2,030.03	1,894.53	1,747.79	1,603.61	-	-	98.31	91.75	84.64	77.66	-	-
up to 580	60.59	21.02	−0.000675	10,516.23	2,064.39	2,026.95	1,891.86	1,746.07	1,602.94	-	-	98.19	91.64	84.58	77.65	-	-
up to 610	60.40	21.02	−0.000669	10,953.87	2,065.19	2,027.59	1,893.56	1,748.91	1,606.83	1,471.72	-	98.18	91.69	84.69	77.81	71.26	-
up to 640	60.30	21.02	−0.000670	11,363.46	2,064.98	2,027.12	1,893.02	1,748.35	1,606.27	1,471.18	-	98.17	91.67	84.67	77.79	71.24	-
up to 671	60.59	21.02	−0.000675	11,764.02	2,064.59	2,027.15	1,892.05	1,746.24	1,603.10	1,467.07	-	98.19	91.64	84.58	77.65	71.06	-
up to 701	62.35	21.04	−0.000674	12,171.38	2,064.50	2,031.52	1,896.62	1,750.14	1,606.04	1,469.00	1,340.76	98.40	91.87	84.77	77.79	71.16	64.94
up to 732	64.29	21.05	−0.000684	12,530.69	2,062.22	2,033.74	1,896.87	1,747.45	1,600.35	1,460.56	1,329.93	98.62	91.98	84.74	77.60	70.82	64.49

t, day of lactation; y_m_, maximum daily milk yield during lactation; r, coefficient describing the rate of decline of the lactation curve between its top and end.
